# Design and Test of Adaptive Leveling System for Orchard Operation Platform

**DOI:** 10.3390/s25051319

**Published:** 2025-02-21

**Authors:** Jianpeng Guo, Zemin Lu, Bingbo Cui, Yuanzhen Xie

**Affiliations:** 1 Key Laboratory of Modern Agricultural Equipment and Technology, Jiangsu University, Ministry of Education, Zhenjiang 212013, China; 2212216050@stmail.ujs.edu.cn (J.G.); cuibingbo@ujs.edu.cn (B.C.); 2222316029@stmail.ujs.edu.cn (Y.X.); 2 School of Agriculture Engineering, Jiangsu University, Zhenjiang 212013, China

**Keywords:** orchard operation platform, fuzzy PID, adaptive leveling

## Abstract

When the orchard operation platform is in use within the orchard, issues of tilting and overturning can arise due to uneven ground, necessitating instant leveling. In this study, the orchard operation platform is simplified into a four-point leveling mechanism, and an adaptive leveling system based on an inertial measurement unit (IMU) is designed. The relationship between coordinate transformation is utilized to derive the platform tilt angle and the position error relationship of the electric actuator, allowing for the analysis of the angle adjustment factors of the leveling mechanism. Through co-simulation using MATLAB and ADAMS, fuzzy control is implemented in addition to PID control, resulting in improved performance. A prototype model of the orchard operation platform is produced and tested, with the platform’s attitude angle remaining stable within a range of ±1.5°. The average leveling time is found to be within 3.6 s. The mean values of dynamic leveling inclination under PID and fuzzy PID control are 2.6° and 1.6°, respectively, with corresponding standard deviations of 1.4° and 0.8°. It conforms to the development trend of agricultural machinery electrification and intelligence and provides a reference basis for manufacturers.

## 1. Introduction

China’s fruit planting area spans approximately 160 million acres annually, encompassing a vast expanse of land [1,2]. However, much of this area is characterized by hilly and mountainous regions with complex terrain, which poses significant challenges for agricultural machinery operations [3,4,5]. Tasks such as pruning, flower thinning, fruit thinning, bagging, picking, and transportation in orchards heavily depend on manual labor, resulting in low efficiency [6,7,8]. As an important auxiliary tool, fruit farmers are gradually embracing the orchard operation platform. However, conventional orchard operation platforms often encounter issues of tilting and rollover due to uneven ground during operation. Therefore, it is essential to develop an operation platform that is suitable for orchard terrains, capable of maintaining a level position in real-time and ensuring operational safety [9,10,11].

In recent years, numerous experts have focused on the safety of agricultural vehicles operating in orchards. Farzaneh et al. [12] investigated the design and installation methods of Crush Protection Devices (CPD) on all-terrain vehicles, evaluating their safety based on factors such as shape, volume, and installation height to reduce operator injuries in rollover accidents. Zhu Qingyu et al. [13] proposed a prediction method based on multi-sensor fusion to actively detect the attitude of agricultural vehicles. This method employs lidar, an inertial measurement unit, and an encoder to gather terrain information in the vehicle’s path, predict the vehicle’s attitude based on its anticipated position, and calculate the tire contact points. Test results indicate that this method can improve vehicle driving stability to some extent. However, due to the complex and uneven road surfaces encountered during actual orchard operations, designing an adaptive leveling system is crucial for enhancing vehicle driving stability.

Many scholars have conducted corresponding research to mitigate the impact of terrain undulation on mechanical operations. Sun Jingbin et al. [14] designed an attitude adjustment mechanism based on a parallel four-bar mechanism, which was analyzed using finite element analysis in ANSYS software (2019 R2). However, this system could only be adjusted laterally. Salel et al. [15] employed slow active suspension control to reduce body rollover by decreasing the body inclination angle. Hu Lian et al. [16] developed an automatic leveling control system for agricultural machinery that fuses data from accelerometers and gyroscopes to obtain the real-time tilt angle of the tractor, enabling horizontal control of agricultural machinery via an electromagnetic reversing valve. Jiang Yu et al. [17] designed a leveling system for a crawler operating machine, creating an articulated leveling mechanism above the crawler chassis and utilizing hydraulic systems as the leveling power component. The results indicate that the final attitude angle remains within the range of ±1.5°, meeting performance requirements for hilly and mountainous areas. Guo Hui et al. [18] studied a safflower picking robot and designed a four-point supported leveling system, analyzing the theoretical error through MATLAB simulation and demonstrating through experimental tests that the leveling control system can achieve a leveling time within 7 s. According to the literature, some studies of leveling systems remain in the theoretical simulation stage, while others exhibit long leveling response times. Furthermore, the leveling systems designed for agricultural implements or crawler chassis cannot be directly applied to the orchard operation platform, necessitating focused research on the operational characteristics of such platforms. The leveling system for the crawler chassis is typically implemented above the chassis, while the leveling mechanism of the crawler chassis exhibits greater structural complexity [19,20,21]. In contrast, this study employs a wheeled chassis leveling system for the orchard operation platform, offering a simpler structural design compared to tracked chassis leveling configurations.

Therefore, this study designs an adaptive leveling system for orchard operation platforms based on a wheel-legged four-point leveling device. A mathematical model was established through coordinate transformation to calculate the extension distance of each electric actuator. Co-simulation using MATLAB (2021b) and ADAMS (2020) was conducted to verify the feasibility of the leveling theory in a simulated environment. An Inertial Measurement Unit (IMU) was employed to detect the platform’s attitude in real-time. By incorporating fuzzy rules into PID control for control judgments, the retraction of each electric actuator was regulated to adjust the platform’s attitude angles, maintaining operational stability within expected parameters. Finally, the complete adaptive leveling system was integrated into a prototype for experimental validation.

## 2. Materials and Methods

### 2.1. Structure of the Whole Machine

**Figure 1:**
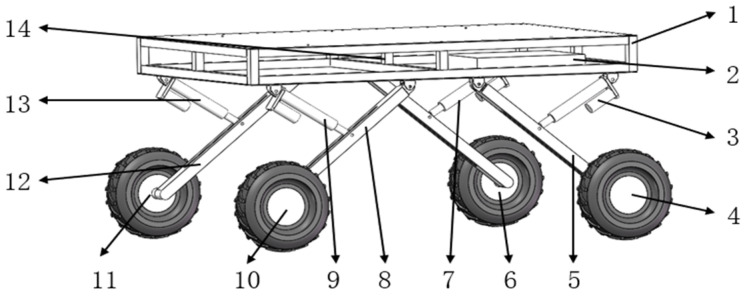
Structure of the whole machine. (1) Chassis. (2) Control unit. (3) Left rear electric actuator. (4) Left rear hub motor. (5) Left rear leg. (6) Right rear hub motor. (7) Right rear electric actuator. (8) Left front leg. (9) Left front electric actuator. (10) Left front hub motor. (11) Right front hub motor. (12) Right front leg. (13) Right front electric actuator. (14) IMU.

The structure of the whole machine is shown in Figure 1; the orchard operation platform is composed of the measurement unit, the leveling unit, the control unit, and the walking unit, in which the measurement unit mainly consists of a 9-degree of freedom patch type IMU, 4 pressure sensors, IMU detects the platform attitude information, while pressure sensors detect each leg pressure to determine whether there is a “virtual leg”. The leveling unit consists of a frame, leg, electric actuator driver and electric actuator. The electric actuator is hinged to both the frame and the leg. The walking unit consists of a motor driver and a hub motor. The motor driver adjusts the speed of the hub motor to fast or slow. The control unit uses STM32F103ZET6 as the main control chip; each unit is connected to the control unit.

The orchard operating platform utilizes four-wheel drive and realizes the forward, backward and steering of the operating platform by driving the hub motor forward and reverse. The control unit realizes automatic leveling by using fuzzy PID to control the expansion and contraction of the electric actuator through the attitude information of the orchard operating platform detected by the IMU.

### 2.2. Leveling Principle of Operation

When the orchard operation platform is driving in orchards, it will be affected by the unevenness of the road surface, which will cause the operation platform to tilt and reduce the safety of fruit farmers’ operation; at this time, it is necessary for the adaptive leveling control system to adjust the attitude of the body of the orchard working platform to the horizontal state. The IMU is installed in the center of the frame and detects the platform’s pitch angle α and roll angle β; the IMU uses serial communication to send signals to the control unit, that is, to acquire the real-time orchard operating platform attitude information. The control unit receives the attitude information and calculates the distance of each electric actuator expansion; the four electric actuators are hinged to the four legs, and the control of the electric actuator expansion makes the angle between the legs and the frame change, which changes the relative height between the platform body and the ground, so as to change the platform body attitude.

**Figure 2:**
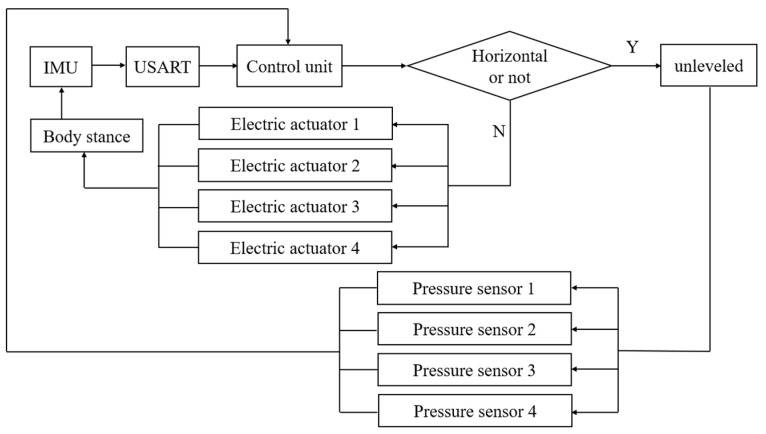
Principle of operation of leveling control.

After leveling, the pressure sensor transmits the detected pressure value to the control unit, which executes a procedure to eliminate any “virtual leg” if detected. The occurrence of a “virtual leg” can be analyzed as follows: multi-point leveling can be categorized into three-point, four-point, and six-point leveling based on the number of legs. While a greater number of legs increases system stability, it also raises the complexity of control accuracy [22,23]. A three-point leveling structure is simpler and easier to control. However, four-point leveling is more prone to generating “virtual legs”, which may result in uneven stress distribution across the platform. This imbalance increases the risk of instability when driving the orchard operating platform. The leveling control principle of the orchard operating platform is illustrated in Figure 2. The control unit receives body attitude information from the platform and sends control signals to regulate the extension and retraction of the four electric push rods in a coordinated sequence. This cyclic process enables closed-loop control, ultimately achieving precise leveling.

### 2.3. Leveling Strategy

#### 2.3.1. Platform Mathematical Modeling

**Figure 3:**
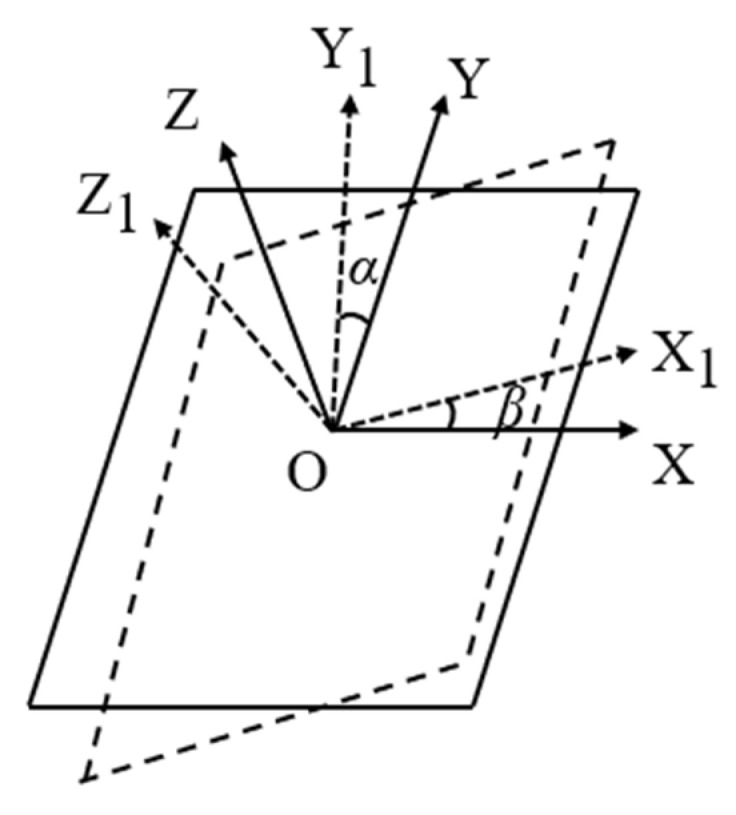
Coordinate transformation diagrams.

Using the IMU installed on the working platform, the pitch angle α and roll angle β of the orchard operating platform are measured. As illustrated in Figure 3, the platform is schematically simplified with the IMU installed at point O, which serves as the coordinate origin of the OXYZ system. The coordinate axes are defined as follows: the Z-axis is oriented vertically upward, the X-axis extends horizontally rightward, and the Y-axis points horizontally forward. Following coordinate rotation, the transformed system $OX_{1}Y_{1}Z_{1}$ is established, with the coordinate transformation relationships depicted in Figure 3. Specifically, the angle between the $Y_{1}$-axis and Y-axis corresponds to the pitch angle α, while the angle between the $X_{1}$-axis and X-axis represents the roll angle β. When both α and β are non-zero, the coordinate transformation matrix for the angle *α* between the $X_{1}$-axis and X-axis is formulated as:

(1) $$R_{1}\left(X,\alpha \right)=\left[\begin{array}{cc} \begin{array}{c} 1\\ 0 \end{array} & \begin{array}{c} \begin{array}{cc} 0 & 0 \end{array}\\ \begin{array}{cc} \cos\alpha & -\sin\alpha \end{array} \end{array}\\ 0 & \begin{array}{cc} \sin\alpha & \cos\alpha \end{array} \end{array}\right]$$

The coordinate transformation matrix for the angle *β* between the $Y_{1}$-axis and Y-axis is formulated as:

(2) $$R_{2}\left(Y,\beta \right)=\left[\begin{array}{cc} \begin{array}{c} \cos\beta \\ 0 \end{array} & \begin{array}{c} \begin{array}{cc} 0 & \sin\beta \end{array}\\ \begin{array}{cc} 1 & 0 \end{array} \end{array}\\ -\sin\beta & \begin{array}{cc} 0 & \cos\beta \end{array} \end{array}\right]$$

The transformation matrix is $R=R_{1}(X,\alpha )R_{2}(Y,\beta )$.

(3) $$R=\left[\begin{array}{cc} \begin{array}{cc} \cos\beta & 0 \end{array} & \sin\beta \\ \begin{array}{cc} \begin{array}{c} \sin\alpha \sin\beta \\ -\cos\alpha \sin\beta \end{array} & \begin{array}{c} \cos\alpha \\ \sin\alpha \end{array} \end{array} & \begin{array}{c} -\sin\alpha \cos\beta \\ \cos\alpha \cos\beta \end{array} \end{array}\right]$$

Since *α* and *β* are not equal to 0 and are small, the expansion according to Taylor’s formula leads to sinα≈α, sinβ≈β, cosα≈cosβ≈1, so the transformation matrix R can be simplified as:

(4) $$R=\left[\begin{array}{cc} \begin{array}{c} 1\\ 0 \end{array} & \begin{array}{c} \begin{array}{cc} 0 & \beta \end{array}\\ \begin{array}{cc} 1 & -\alpha \end{array} \end{array}\\ -\beta & \begin{array}{cc} \alpha & 1 \end{array} \end{array}\right]$$

In the frame space coordinate system, it is assumed that the coordinates $P_{i1}$ of each motorized actuator and frame articulation point are:

(5) $$P_{i1}=\left(X_{i1},Y_{i1},Z_{i1}\right)$$

where i takes the values 1, 2, 3, 4.

According to Equations (4) and (5), it can be deduced that the coordinates $P_{i}$ of each motorized actuator and frame articulation point under the spatial coordinate system in the horizontal plane are

(6) $$P_{i}=\left[\begin{array}{cc} \begin{array}{c} 1\\ 0 \end{array} & \begin{array}{c} \begin{array}{cc} 0 & \beta \end{array}\\ \begin{array}{cc} 1 & -\alpha \end{array} \end{array}\\ -\beta & \begin{array}{cc} \alpha & 1 \end{array} \end{array}\right]\left[\begin{array}{c} X_{i1}\\ Y_{i1}\\ Z_{i1} \end{array}\right]$$

where i takes the values 1, 2, 3, 4.

The error leveling method can be divided into angle error leveling and position error leveling. Position error leveling is typically used in scenarios that require high control accuracy, whereas angle error leveling has shorter leveling times but lower accuracy compared to position error leveling. Position error leveling was selected in this study to ensure the accuracy of orchard leveling. In this method, the position error that needs adjustment is calculated using the attitude angle of the orchard operating platform’s body. Position error leveling is further divided into three types: chasing type, center point immobility, and set point immobility. Among these, the center point immobility method is less time-consuming. Considering that the orchard operation platform needs to achieve efficient and fast leveling during both driving and stationary states, the center point immobility method was chosen. This method maintains the height of the geometric center point of the load platform’s leveling plane while using the frame’s center point as a reference. Other points on the platform are adjusted so that they, along with the frame’s center point, lie on the same horizontal plane. The Z-axis coordinates of each electric push rod and the frame’s hinge point can be calculated using Equation (7).

(7) $$Z_{i}=\left[\begin{array}{ccc} -\beta & \alpha & 1 \end{array}\right]\left[\begin{array}{c} X_{i}\\ Y_{i}\\ Z_{i} \end{array}\right]$$

**Figure 4:**
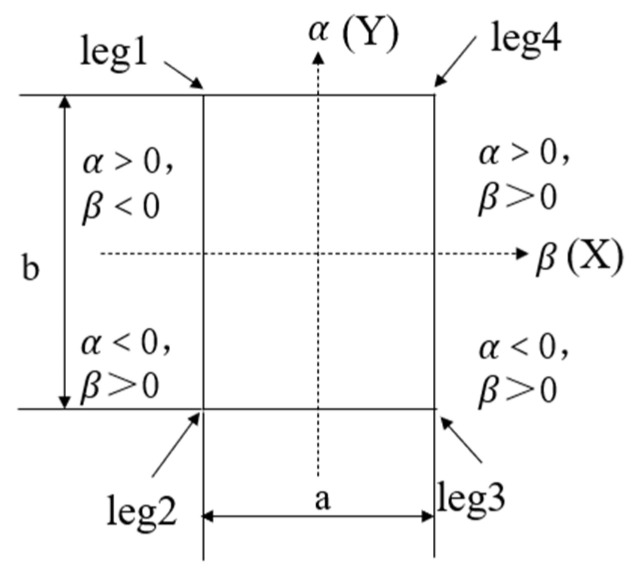
Leveling divisions.

The leveling division of the orchard operation platform shown in Figure 4 has the following pitch angle α and traverse roll angle β.

Assuming the initial state of the orchard operation platform is as shown in Figure 4, where “α>0” and “β>0”, the length and width of the platform are denoted as “b” and “a”, respectively. By taking the center point of the frame as the origin and using the length and width of the platform as the two axes, a two-dimensional coordinate system is established. The position errors are relative to the center point, namely “$e_{1}$”, “$e_{2}$”, “$e_{3}$”, and “$e_{4}$”, can be derived from Equation (7).

(8) $$\left\{\begin{array}{c} e_{1}=\frac{a}{2}\beta +\frac{b}{2}\alpha \\ \begin{array}{c} e_{2}=\frac{a}{2}\beta -\frac{b}{2}\alpha \\ \begin{array}{c} e_{3}=-\frac{a}{2}\beta -\frac{b}{2}\alpha \\ e_{4}=-\frac{a}{2}\beta +\frac{b}{2}\alpha \end{array} \end{array} \end{array}\right.$$

The error obtained from Equation (8) is used as the input signal for fuzzy PID control. The output control signal regulates the expansion and contraction of the electric actuator. The larger the error, the faster the electric actuator expands or contracts, enabling the side with the greater error to reduce it quickly. IMU continuously detects the attitude information of the orchard operation platform, updating the error in real-time. The leveling cycle is repeated until the leveling requirements are met.

#### 2.3.2. Analysis of Leveling Structure and Leveling Maximum Tilt Angle

**Figure 5:**
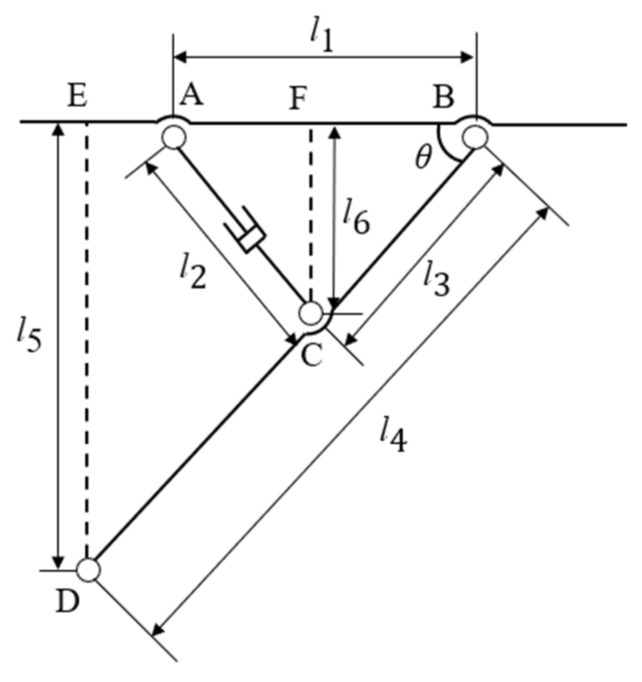
Orchard operation platform leveling structure.

The leveling structure of the orchard operation platform is divided into four groups. Each group consists of a leg, an electric actuator, and a frame. The leg and the frame are hinged, the electric actuator and the frame are hinged, and the electric actuator and the leg are also hinged. These four groups are symmetrically distributed around the center of the frame. The leveling mechanism of each group is shown in Figure 5; when the forward speed of the platform is not considered, the movement of the legs is planar rotation. On encountering an uneven road surface, the electric actuator controls the telescopic motion, driving the leg to rotate around the hinge point between the leg and the frame. This adjusts the angle between the leg and the frame, altering the platform’s height off the ground to ensure that the orchard operation platform remains level. In Figure 5, AB is the distance between two points where the leg and the frame are articulated and the electric actuator and the frame are hinged, which is expressed by $l_{1}$, AC is the length of the electric actuator, assuming that the electric actuator is contracted to the lowest at this moment, which is expressed by $l_{2}$, BD is the length of the hinged, which is expressed by $l_{4}$, and the distance between BC is expressed by $l_{3}$, DE, CF is perpendicular to the line between the two points AB, the distance between DE is represented by $l_{5}$, the distance between CF is represented by $l_{6}$, through the derivation can be derived from the relationship between the length of the electric actuator AC and the angle θ between the legs and frame:

(9) $$l_{6}/l_{5}=l_{3}/l_{4}$$

(10) $$\sin\theta =\frac{l_{6}}{l_{3}}=\frac{l_{5}}{l_{4}}$$

By the cosine theorem:

(11) $$l_{2}=\sqrt{{l_{1}}^{2}+{l_{3}}^{2}-2l_{1}\cdot l_{3}\cos\theta }$$

Combining Equations (10) and (11):

(12) $$\theta =\arcsin\sqrt{1-\frac{{l_{1}}^{2}+{l_{3}}^{2}-{l_{2}}^{2}}{4{l_{1}}^{2}\cdot {l_{3}}^{2}}}$$

**Figure 6:**
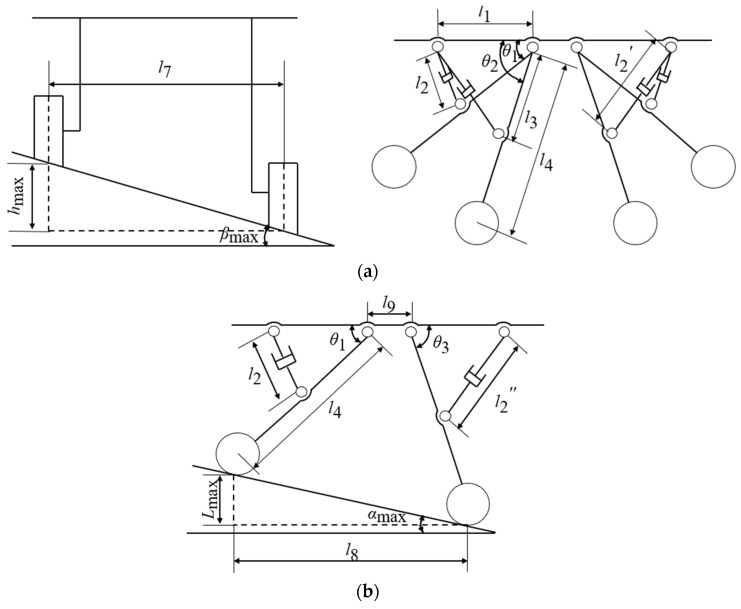
Maximum tilting angle of orchard operation platform. (**a**) Maximum lateral inclination. (**b**) Maximum longitudinal inclination.

When the leveling mechanism on one side of the orchard operation platform is lowered to its minimum position while the leveling mechanism on the other side is adjusted to its maximum height, the maximum cross-roll angle is reached, as illustrated in Figure 6a. The maximum cross-roll angle of this orchard operation platform can be calculated as follows:

(13) $$h_{\max}=l_{4}\times \left(\sin\theta_{2}-\sin\theta_{1}\right)$$

The lengths of $l_{1}$ and $l_{3}$ are kept constant in Figure 5. By combining Figure 5 with Equation (12), we obtain:

(14) $$h_{\max}=l_{4}\times (\sqrt{1-\frac{{l_{1}}^{2}+{l_{3}}^{2}-{l_{2}}^{\prime 2}}{4{l_{1}}^{2}\cdot {l_{3}}^{2}}}-\sqrt{1-\frac{{l_{1}}^{2}+{l_{3}}^{2}-{l_{2}}^{2}}{4{l_{1}}^{2}\cdot {l_{3}}^{2}}})$$

(15) $$\beta_{\max}\approx \arctan\frac{h_{\max}}{l_{7}}$$

where $\beta_{\max}$ is the maximum cross-roll angle of the orchard operation platform; $h_{\max}$ is the maximum lifting height of the orchard operation platform; $l_{7}$ is the wheelbase of the orchard operation platform, which is approximately equal to the width of the frame; and $\theta_{1}$ and $\theta_{2}$ are the angles between the legs and the frame.

When the leveling mechanism on either the front or rear side of the orchard operation platform is lowered to its lowest position, while the leveling mechanism on the opposite side is adjusted to its maximum height, the maximum pitch angle can be obtained, as shown in Figure 6b. The maximum pitch angle of this orchard operation platform is calculated as follows:

(16) $$L_{\max}=l_{4}\times \left(\sin\theta_{3}-\sin\theta_{1}\right)$$

The lengths of $l_{1}$ and $l_{3}$ are kept constant in Figure 5. By combining Figure 5 with Equation (12), we obtain:

(17) $$L_{\max}=l_{4}\times \left(\sqrt{1-\frac{{l_{1}}^{2}+{l_{3}}^{2}-{l_{2}}^{\prime \prime 2}}{4{l_{1}}^{2}\cdot {l_{3}}^{2}}}-\sqrt{1-\frac{{l_{1}}^{2}+{l_{3}}^{2}-{l_{2}}^{2}}{4{l_{1}}^{2}\cdot {l_{3}}^{2}}}\right)$$

(18) $$\alpha_{\max}\approx \arctan\frac{L_{\max}}{l_{4}\times \left(\frac{{l_{1}}^{2}+{l_{3}}^{2}-{l_{2}}^{\prime \prime 2}}{4{l_{1}}^{2}\cdot {l_{3}}^{2}}+\frac{{l_{1}}^{2}+{l_{3}}^{2}-{l_{2}}^{2}}{4{l_{1}}^{2}\cdot {l_{3}}^{2}}\right)+l_{9}}$$

where $\alpha_{\max}$ is the maximum pitch angle of the orchard operation platform; $L_{\max}$ is the maximum lifting height of the orchard operation platform; $l_{4}$ is the leg length; $l_{9}$ is the distance between the two legs on the same side and the hinge point of the frame; and $\theta_{1}$ and $\theta_{3}$ are the angles between the legs and the frame.

### 2.4. Control System Design

#### 2.4.1. Hardware Design

**Figure 7:**
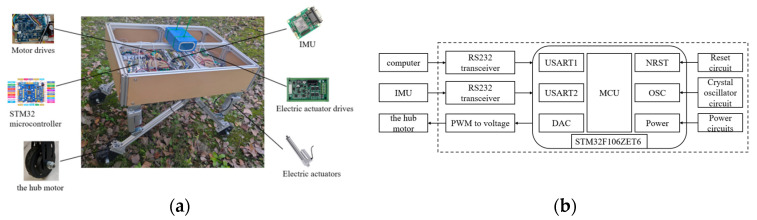
Adaptive leveling system components. (**a**) Hardware composition. (**b**) System Architecture Framework.

The adaptive leveling system of the orchard operation platform consists of a mobile platform, a control unit, wheel motors, wheel motor drivers, electric actuators, an IMU, pressure sensors, electric actuator controllers, and a serial communication module. The adaptive leveling control system is installed on the orchard operation platform. The hardware composition of the adaptive leveling system is shown in Figure 7a, while Figure 7b illustrates the system structure framework. The control unit in the adaptive leveling control system utilizes the STM32F103ZET6 microcontroller produced by Guangzhou Xingyi Electronic Technology Co., Ltd. (Guangzhou, China). The IMU selected is the 9-axis CH040 SMD attitude sensor from Beijing Supercore Electronics Co., Ltd. (Beijing, China), which has a transmission signal frequency of 100 Hz and can achieve static leveling within 0.05° and dynamic leveling within 0.1°. The drive motor is a 24 V hub motor, with speed adjusted via the motor driver. The rated voltage of the motor driver is 24 V, which is grounded with the STM32 microcontroller. The STM32 microcontroller outputs an analog voltage ranging from 0 to 5 V, which is transmitted to the motor controller to control the speed of the hub motor; the larger the analog voltage, the faster the hub motor operates. The leveling mechanism employs an electric push rod with a rated voltage of 12 V and a rated speed of 55 mm/s. However, the electric actuator cannot be directly controlled by the control unit; it requires cooperation with the model YF-80 electric actuator driver, which has a rated voltage of 12 V and a rated output current of 7 A. The control of the electric actuator’s extension and contraction is accomplished by generating two pulse signals through the control unit, which are sent to the electric actuator controller. The first pulse signal represents the extension of the electric actuator, while the second represents its contraction. In the program, the extension of the electric actuator is denoted as ‘10’, and the contraction is denoted as ‘01’. The electric actuator driver converts the received pulse signal into an electrical signal to control the expansion and contraction of the electric actuator. Additionally, a laptop serves as a serial assistant to print the data received by the STM32 microcontroller.

**Figure 8:**
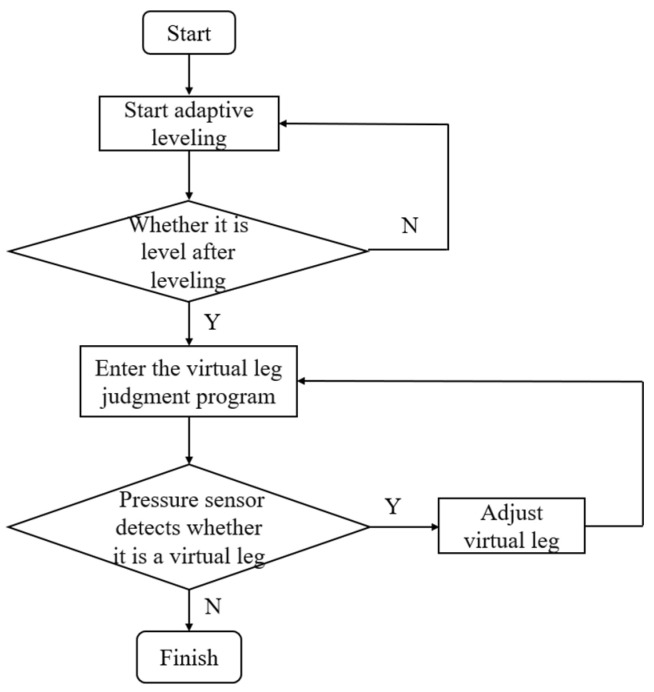
Virtual leg control flow.

In this study, pressure sensors are installed at the connection between the electric actuators and the legs to detect the pressure on the electric actuators. A closed-loop control strategy is adopted to effectively address the issue of the “virtual leg”. The control unit must evaluate the value of $p_{i}$ (where *i* = 1,2,3,4) transmitted by each pressure sensor to determine whether each leg is grounded and load-bearing, based on the pressure value calculated when p represents the self-weight of the orchard operation platform. By using the condition $p_{i}\leq p$ (where p is the pressure value calculated for the self-weight of the orchard operation platform), the unit assesses whether each leg is grounded to bear weight, ensuring the safety of the orchard operation platform. The detection of the “virtual leg” is illustrated in Figure 8.

#### 2.4.2. Fuzzy PID Controller Design

Adaptive leveling of the orchard operation platform is a crucial technology for achieving high-efficiency operation and enhancing driving safety. While PID parameters are typically set as fixed values, which limits the potential benefits of parameter tuning [24,25], the introduction of fuzzy PID control provides an effective solution for the leveling of orchard operation platforms.

PID control algorithm is

(19) $$u\left(t\right)=K_{p}e\left(t\right)+K_{i}\int_{0}^{t}e\left(t\right)\mathrm{dt}+K_{d}\frac{\mathrm{de}\left(t\right)}{\mathrm{dt}}$$

where u(t) is the output; e(t) is the input; $K_{p}$ is the proportional gain; $K_{i}$ is the integral gain; $K_{d}$ is the differential gain.

**Figure 9:**
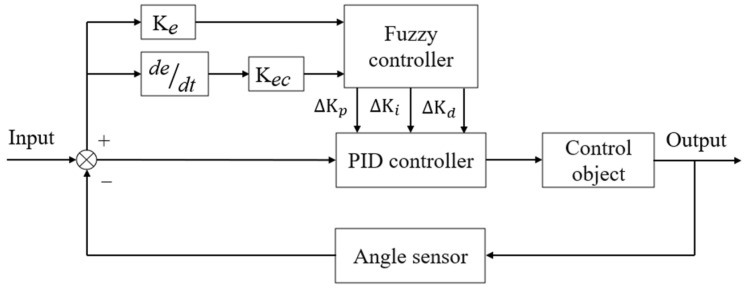
Fuzzy controller.

Fuzzy PID control comprises fuzzification, fuzzy inference, and defuzzification processes [26,27]. The error e and the rate of change of the error $e_{c}$ serve as the inputs to the fuzzy controller. After fuzzification, the corresponding linguistic variables E and $E_{c}$ are obtained. The fuzzy controller then executes fuzzy inference and defuzzification operations on the derived E and $E_{c}$. The fuzzy rule table is established based on expert experience and relevant knowledge [28,29,30,31,32]. The fuzzy controller outputs the variation amounts for real-time adjustment of the three PID controller parameters $K_{p}$, $K_{i}$, and $K_{d}$, represented as $∆K_{p}$, $∆K_{i}$, and $∆K_{d}$, respectively. The fuzzy controller is illustrated in Figure 9.

(20) $$\left\{\begin{array}{c} K_{p}=K_{p0}+∆K_{p}\\ K_{i}=K_{i0}+∆K_{i}\\ K_{d}=K_{d0}+∆K_{d} \end{array}\right.$$

The feasible intervals for the leveling error e and the rate of change of the leveling error $e_{c}$ are set to [−10 cm, 10 cm] and [−5.5 cm/s, 5.5 cm/s], respectively. The feasible intervals for $∆K_{p}$ are set to [−3, 3], for $∆K_{i}$ to [−1, 1], and for $∆K_{d}$ to [−3, 3]. The membership functions for all parameters are defined using trigonometric subordination functions. The fuzzy conditional statements are represented by the following set of fuzzy terms: NB (Negative Big), NM (Negative Medium), NS (Negative Small), ZO (Zero), PS (Positive Small), PM (Positive Medium), and PB (Positive Big). The fuzzy subsets of the input and output variables are quantized into seven levels, denoted as [NB, NM, NS, ZO, PS, PM, PB], with the basic thesis domain set to [−6, 6]. In this study, it is considered that the tuning of the PID parameters, as well as the realization of a rapid system response, should be based on the following rules:

(1) When the deviation is large, to eliminate the deviation as quickly as possible and improve the response speed, $K_{p}$ should be increased. To prevent overshooting of the system, it is necessary to increase $K_{p}$ while reducing $K_{d}$, and $K_{i}$ should be set to zero or a very small value.
(2) When the deviation is small, to continue eliminating the deviation while simultaneously reducing oscillation and preventing overshooting, $K_{i}$ should be increased and $K_{p}$ should be appropriately decreased.
(3) When the rate of change of the deviation is large, $K_{p}$ should be reduced, and $K_{i}$ should be increased.
(4) The steady-state performance of the system can be adjusted through the differential component. When the error is very large, the value of $K_{d}$ should be set to zero or a very small value to reduce oscillation. Based on the above four rules, the fuzzy control rules for $∆K_{p}$, $∆K_{i}$, and $∆K_{d}$ are established.

The fuzzy rule table is as shown in Table 1, Table 2 and Table 3.

### 2.5. Co-Simulation Design Based on MATLAB-ADAMS

#### 2.5.1. Co-Simulation Model Design

**Figure 10:**
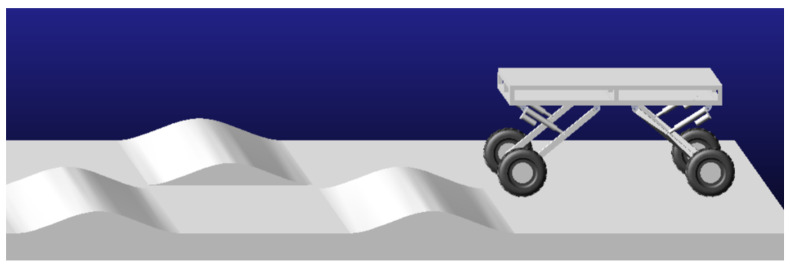
Continuous undulating slopes.

The 3D modeling software SolidWorks (2022) was utilized to create the 3D model of the orchard operation platform, which was subsequently imported into ADAMS. After simplifying the model, adding constraints, assigning materials, and setting contact parameters, the road surface for the orchard operation platform was simulated. The road surface model, illustrated in Figure 10, simulates the working conditions of the orchard operation platform. It consists of a continuous undulating slope with a wavelength of 1700 mm, a wave crest of 150 mm, and a slope of 10°.

#### 2.5.2. Co-Simulation System Construction

**Figure 11:**
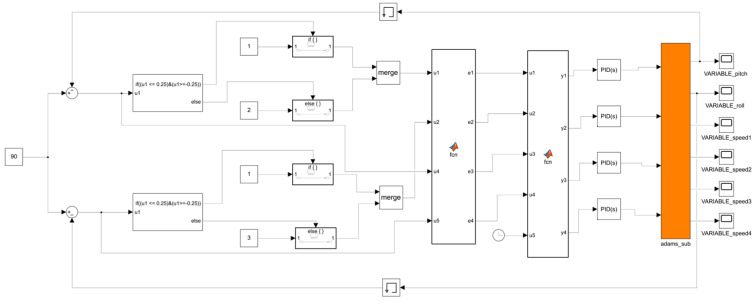
PID control system model in Simulink.

**Figure 12:**
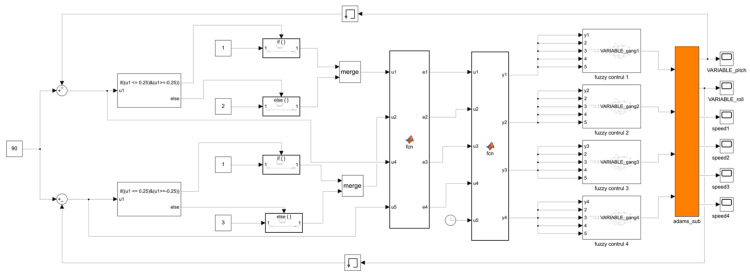
Fuzzy PID control system model in Simulink.

**Figure 13:**
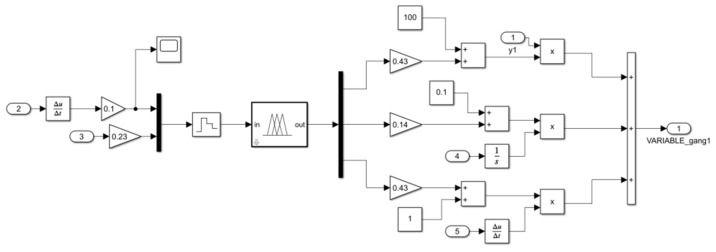
Fuzzy PID controller module.

Based on the leveling working principle, leveling strategy, and controller design described above, a control system for MATLAB-ADAMS co-simulation was established [33,34]. In this study, two methods will be employed to control the system: PID and fuzzy PID. The simulation models were constructed in MATLAB/Simulink (2021b), as depicted in Figure 11 and Figure 12. Figure 11 presents the PID control system model, while Figure 12 illustrates the fuzzy PID control system model. The fuzzy PID controller is detailed in Figure 13. Both PID and fuzzy PID control system models receive various parameters output by the ADAMS system module as inputs, specifically the pitch angle and roll angle, which are fed back into the system. PID controller or fuzzy PID controller then transforms these inputs into four electric actuator extension speed control variables for the leveling mechanism of the orchard operation platform. These control variables are transmitted to the input of the ADAMS system module, serving as the output of the control system in the simulation model. Additionally, the ADAMS system module implements closed-loop control of the attitude information feedback from the output of the orchard operation platform.

#### 2.5.3. Co-Simulation Results Analysis

**Figure 14:**
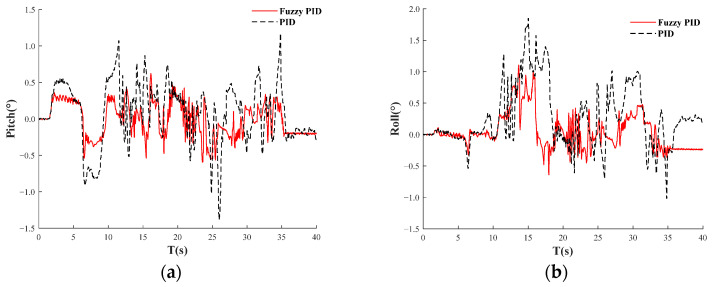
Comparison of co-simulation results. (**a**) Comparison of PID and fuzzy PID leveling control pitch angles. (**b**) Comparison of PID and fuzzy PID leveling control roll angles.

In the MATLAB-ADAMS co-simulation, the road surface conditions are considered ideal, with a leveling threshold set at ±0.5°. Figure 14a presents a comparison of pitch angle control between PID and fuzzy PID dynamic leveling, while Figure 14b shows the comparison of roll angle control using the same methods. The total simulation duration is 40 s. Analysis of the simulation results indicates that the overall leveling performance of fuzzy PID control surpasses that of PID control. Specifically, the pitch angle range for PID control is confined to ±1.5°, whereas fuzzy PID control achieves a pitch angle range of ±0.5°. Similarly, the roll angle for PID control is limited to ±2°, while fuzzy PID control demonstrates a roll angle range of ±1°. In the adaptive leveling system, the maximum pitch angle controlled by PID is −1.4°, while the maximum roll angle is 1.8°. In contrast, the maximum pitch angle controlled by fuzzy PID is 0.6°, and the maximum roll angle is 1.1°. This paper evaluates dynamic test parameters, including the mean value of inclination and the standard deviation of inclination. The inclination of the orchard operation platform is derived from the pitch angle and roll angle. The calculation formula is as follows:

(21) $$\gamma_{k}=\sqrt{\alpha_{k}^{2}+\beta_{k}^{2}}$$

where $\alpha_{k}$ is the pitch angle of the orchard operation platform at time *k*; $\beta_{k}$ is the roll angle of the orchard operation platform at time *k*; $\gamma_{k}$ is the inclination of the orchard operation platform at time *k*.

The formula for calculating the evaluation parameters of dynamic adjustment is as follows:

(22) $$\overset{-}{\gamma }=\frac{1}{N}\sum_{K=0}^{N}\gamma_{k}$$

(23) $$S_{\gamma }=\sqrt{\frac{1}{N}\sum_{k=0}^{N}{\left(\gamma_{k}-\overset{-}{\gamma }\right)}^{2}}$$

where $\overset{-}{\gamma }$ is the mean value of inclination; $S_{\gamma }$ is the standard deviation of inclination; N is the total number of points recorded in a single test.

The calculation indicates that the average inclination of the orchard operation platform under PID control is 0.6°, with a standard deviation of 0.4°. In contrast, the average inclination under fuzzy PID control is 0.3°, accompanied by a standard deviation of 0.2°. Notably, all evaluation parameters for fuzzy PID control are lower than those for PID control, reflecting a 33% reduction in both the mean slope value and the standard deviation. Furthermore, as illustrated in Figure 14, the dynamic leveling amplitude exhibits minimal variation during fuzzy PID control, suggesting that fuzzy PID control achieves superior dynamic leveling performance and a faster response.

The calculation indicates that the average inclination of the orchard operation platform under PID control is 0.6°, with a standard deviation of 0.4°. In contrast, the mean value of inclination under fuzzy PID control is 0.3°, accompanied by a standard deviation of 0.2°. Notably, all evaluation parameters for fuzzy PID control are lower than those for PID control, reflecting a 33% reduction in both the mean value of inclination and the standard deviation. Furthermore, as illustrated in Figure 14, the dynamic leveling amplitude exhibits minimal variation during fuzzy PID control, suggesting that fuzzy PID control achieves superior dynamic leveling performance and a faster response.

## 3. The Experimental Program and Analysis of Results

### 3.1. The Experimental Program

**Figure 15:**
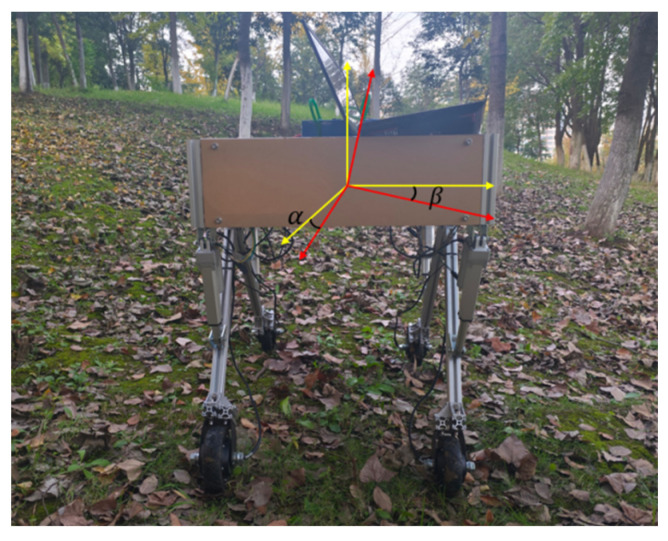
The on-site testing of the orchard operation platform.

The experimental program is divided into two scenarios: static test and dynamic test. Considering the impact of terrain undulations during operation, the leveling threshold is established at ±1.5° after conducting multiple tests, with no leveling adjustments made within this range. The static test primarily assesses the leveling effect of the orchard operation platform in response to sudden changes in the road, while the dynamic test simulates the leveling effect of the platform under actual driving conditions. To verify the feasibility of the adaptive leveling system, a test was conducted on the orchard operation platform. Figure 15 illustrates the on-site testing situation of the orchard operation platform, where α represents the pitch angle and β denotes the roll angle.

**Figure 16:**
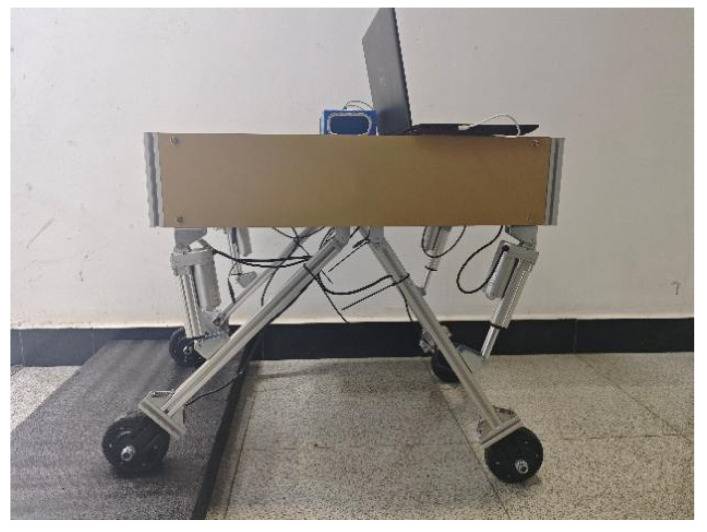
The on-site static test.

Static Test: the attitude information of the orchard operation platform is detected using IMU, specifically measuring the pitch and roll angles. This data is transmitted to a serial port assistant via serial communication, and the leveling time is recorded. Based on the co-simulation results, the performance of fuzzy PID control for leveling is superior to that of PID control. Consequently, the static leveling test employs fuzzy PID automatic leveling, allowing for manual adjustments to the inclination of the orchard operation platform (The tilt angle for each test group was selected not to exceed the maximum adjustment angle of 10°), positioning it at the highest front and rear points. There are eight distinct states to consider: front highest, rear highest, left highest, right highest, left front highest, left rear highest, right rear highest, and right front highest. The evaluation parameters for static leveling performance include adjustment time and tilt adjustment error. The results of the static field test are illustrated in Figure 16.

The formula for calculating the evaluation parameters of static leveling is as follows:

(24) $$\gamma_{e}=\frac{\sum_{k=k_{e}}^{k_{e}+20}\gamma_{k}}{20}$$

where $k_{e}$ is the number of sample points recorded at the pitch and roll angles when the system adjustment is completed; $\gamma_{e}$ is inclination adjustment error.

**Figure 17:**
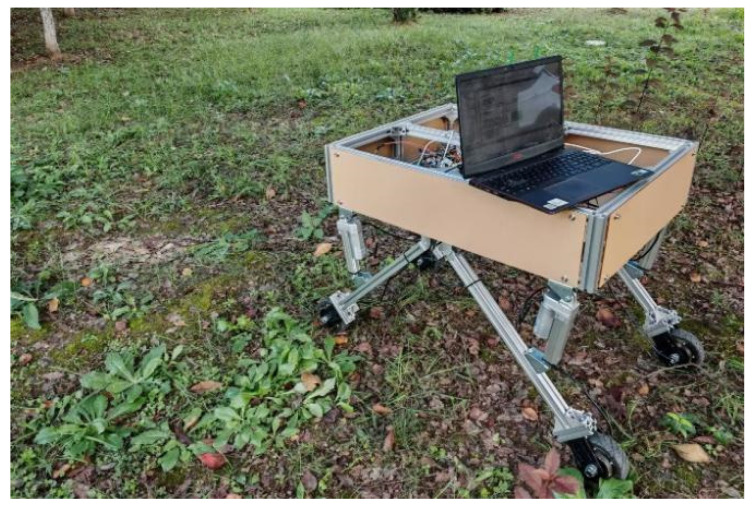
The on-site dynamic test.

Dynamic test: an uneven road surface is selected at the test base. Prior to the test, both the starting and ending points of the road surface are calibrated, and the adaptive leveling system is deactivated. The orchard operation platform is then operated at a speed of 1.8 km/h. Road conditions are detected using IMU, with the data transmitted via serial communication to the STM32 microcontroller for processing. The attitude information of the orchard operation platform, specifically the pitch and roll angles, is represented in a curve. Next, the orchard operation platform is repositioned at the starting point of the road, the adaptive leveling system is reactivated, and the platform is driven at the same speed as in the previous path. The pitch and roll angle information are recorded and represented in a curve, as illustrated in Figure 19. The on-site dynamic test is depicted in Figure 17. The adaptive leveling system employs both PID and fuzzy PID for control. The evaluation parameters for dynamic leveling performance include the mean and standard deviation of the inclination, as referenced in Equations (21)–(23). In dynamic testing, the effectiveness of leveling is influenced not only by the leveling algorithm and control system but also by the driving speed. For the orchard operation platform, the driving speed should not exceed 1.8 km/h, which is established as the maximum operational speed in such environments. Exceeding this speed threshold necessitates re-evaluating the operational parameters of the electric actuator to maintain optimal system performance.

### 3.2. Analysis of Results

#### 3.2.1. Analysis of Static Test Results

**Figure 18:**
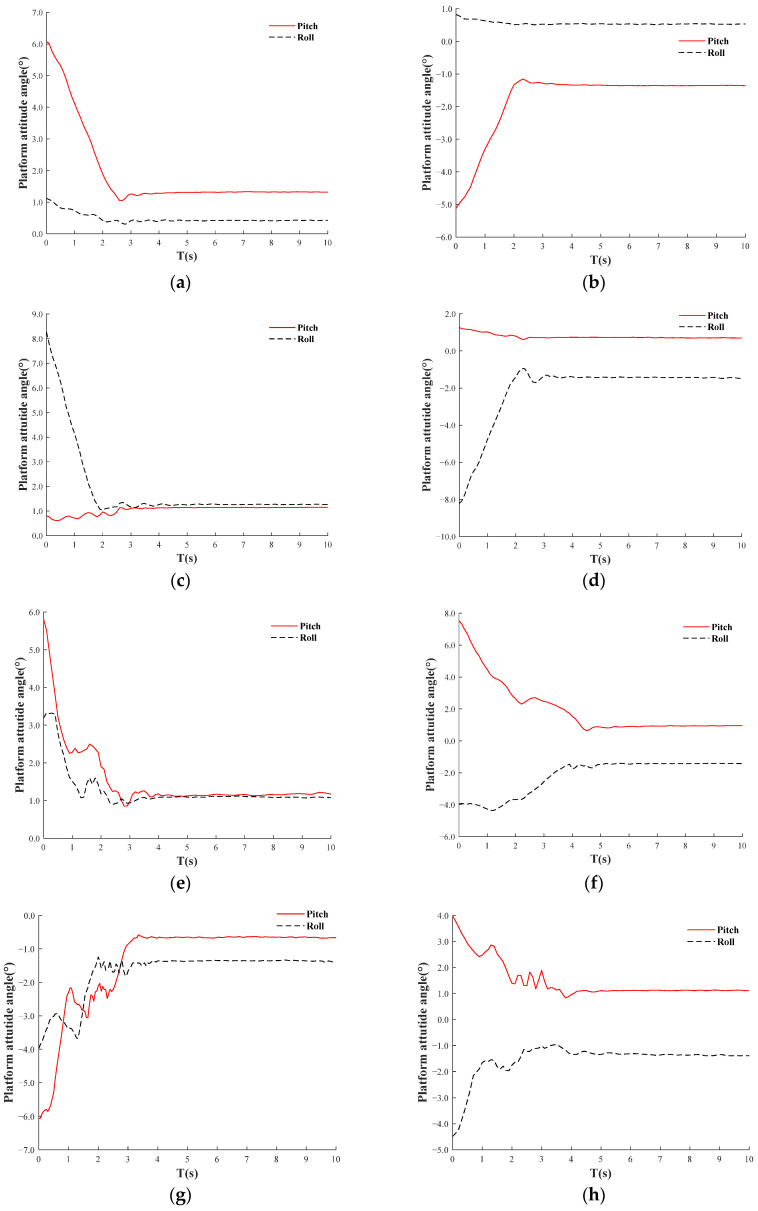
Adaptive leveling of results in eight static tilt states. (**a**) Front highest, (**b**) Rear highest, (**c**) Left highest, (**d**) Right highest, (**e**) Left front highest, (**f**) Left rear highest, (**g**) Right rear highest, (**h**) Right front highest.

Figure 18 displays the results of adaptive leveling across eight static tilt states based on fuzzy PID control, visually illustrating the leveling process. In the four simple pitch and roll angle adjustments, namely front highest, rear highest, left highest, and right highest, adaptive leveling time is generally within 3 s. In contrast, for the four mixed pitch and roll angle adjustments, namely left front highest, left rear highest, right rear highest, and right front highest, the adaptive leveling time is typically within 4.7 s, with minimal overshoot observed during the adaptive leveling process across all eight tilt states. Table 4 provides a clearer representation of the attitude angle variations before and after leveling. The initial inclinations for these eight states are calculated as follows: 6.2°, 5.2°, 8.1°, 8.3°, 6.6°, 8.7°, 7.3°, and 6.1°. The corresponding adjustment times are 3.0 s, 2.8 s, 3.0 s, 3.1 s, 3.8 s, 4.7 s, 3.7 s, and 4.5 s. The inclination adjustment errors are 1.3°, 1.4°, 1.6°, 1.5°, 1.5°, 1.7°, 1.6°, and 1.6°. As demonstrated in Table 4 and Figure 18, the leveling results have been maintained within the specified range of ±1.5°, achieving the expected leveling performance. The shortest leveling time recorded is 2.8 s, while the maximum is 4.7 s, yielding an average adjustment time of 3.6 s. The maximum inclination adjustment error is 1.7°, the minimum is 1.3°, and the average is 1.5°, with no significant overshoot or oscillation observed throughout all adjustment processes.

#### 3.2.2. Analysis of Dynamic Test Results

**Figure 19:**
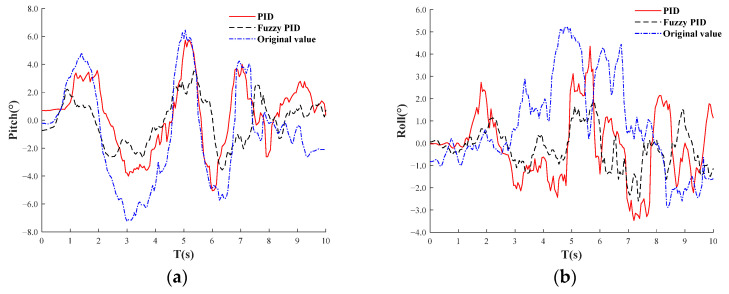
Dynamic test results. (**a**) PID-adjusted pitch angle, fuzzy PID-adjusted pitch angle, and the original pitch angle curve of the road surface. (**b**) PID-adjusted roll angle, fuzzy PID-adjusted roll angle, and the original roll angle curve of the road surface.

The results of the dynamic test are shown in Figure 19. Figure 19a displays PID-adjusted pitch angle, fuzzy PID-adjusted pitch angle, and the original pitch angle curve of the road surface, while Figure 19b presents PID-adjusted roll angle, fuzzy PID-adjusted roll angle, and the original roll angle curve of the road surface. Under fuzzy PID control, the dynamic adjustment pitch angle range of the orchard operation platform is within ±4°, and the roll angle range is within ±3°, ultimately stabilizing within ±1.5°. According to the obtained data, the average leveling inclination of the orchard operation platform is 3.8°, with a standard deviation of 2.1°, indicating a significant terrain inclination. When the adaptive leveling is activated, the mean inclination of the orchard operation platform under PID control is 2.6°, and the standard deviation of inclination is 1.4°, both of which are lower than the mean inclination and standard deviation under non-alignment conditions. This suggests that the adaptive leveling system with PID control can improve the inclination state of the orchard operation platform. Under fuzzy PID control, the mean inclination is 1.6°, with a standard deviation of 0.8°. The mean inclination and standard deviation under fuzzy PID control are both smaller than those under PID control, indicating that the adaptive leveling system effectively enhances the inclination stability of the orchard operation platform and improves driving stability. Under the three working conditions, the mean value of the inclination of the orchard platform shows greater variation compared to the standard deviation of inclination, indicating that while the ground is inclined, the fluctuations are minimal. The adaptive leveling system based on fuzzy PID control demonstrates superior leveling performance, providing evidence that it can enhance the adaptability of the orchard operation platform to uneven pavement.

### 3.3. Summary of Test Results

The static and dynamic test results demonstrate that the adaptive leveling system utilizing fuzzy PID control performs exceptionally well. In static tests across eight tilt states, leveling times ranged from 3 s to 4.7 s, with inclination adjustment errors between 1.3° and 1.7°, maintaining results within ±1.5°. During dynamic testing, the system stabilized within ±1.5° under fuzzy PID control. The average leveling inclination of the orchard operation platform was 3.8°, with a standard deviation of 2.1°. When adaptive leveling was engaged, the mean inclination under PID control was 2.6° with a standard deviation of 1.4°, whereas under fuzzy PID control, the mean inclination was reduced to 1.6° with a standard deviation of 0.8°. Across all conditions, the mean inclination varied more than the standard deviation. The adaptive leveling system based on fuzzy PID control offers superior leveling performance, significantly enhancing the platform’s adaptability to uneven terrain.

Table 5 presents a comparison between this study and other research on orchard machinery. In complex orchard terrains, machinery equipped with automatic leveling functions demonstrates superior anti-interference capabilities. Fuzzy PID merges the adaptability of fuzzy logic with the stability of PID, eliminating the need for extensive data training or dependence on the generalization ability of training data. Under identical experimental conditions, fuzzy PID incurs lower costs, affirming its advantages in unstructured environments.

## 4. Conclusions

In this study, an adaptive leveling system based on a four-point leveling method and a center-point immovable leveling method is designed for the orchard operation platform. It is demonstrated through theoretical analysis, modeling, and simulation of the control object that the designed adaptive leveling system can enhance the driving stability of the orchard operation platform.

(1) Theoretical analysis of the orchard operation platform’s leveling structural parameters led to the derivation of a mathematical relationship between the maximum adjustment angle and related parameters. Structural parameters were optimized, and a formula was developed to relate the platform’s attitude angle to the electric actuator’s displacement through coordinate transformation. Furthermore, a center-point leveling strategy was proposed, offering a solid theoretical foundation for leveling simulations and prototype development.
(2) Using MATLAB, the PID algorithm parameters were fine-tuned, and a fuzzy PID algorithm was developed with well-defined fuzzy rules. After extensive testing, optimal parameters for the system were identified. Simulations of the orchard’s uneven terrain were conducted using MATLAB and ADAMS, applying both PID and fuzzy PID control strategies. Results showed that the fuzzy PID algorithm significantly outperformed the traditional PID, maintaining the platform’s attitude angle within the desired range. This finding lays a solid theoretical foundation for the development of the leveling system.
(3) Static and dynamic tests on the prototype revealed an inclination adjustment error of 1.7° and an average leveling time of 3.6 s across eight inclination states. With fuzzy PID control, the prototype’s mean inclination values were 3.8°, 2.6°, and 1.6°, with standard deviations of 2.1°, 1.4°, and 0.8°, respectively. This adaptive leveling system exhibited superior performance, confirming the feasibility and accuracy of the proposed method. The leveling process activates when inclinations exceed ±1.5°, maintaining the platform’s attitude angle within ±3°, thus achieving stable dynamic leveling.
(4) The research findings offer valuable insights for orchard operation platform studies and manufacturers. The theoretical analysis of leveling structural parameters aids in designing prototypes with enhanced leveling performance and understanding the influence of various structural parameters. By adjusting PID parameters and establishing fuzzy rules using MATLAB, followed by co-simulations with ADAMS, manufacturers can adopt the more effective fuzzy PID control algorithm for improved stability on uneven orchard terrain. Additionally, dynamic and static prototype tests provide concrete data on system performance, validating the practical application of the proposed leveling method and guiding the design of reliable and efficient leveling systems.

## Figures and Tables

**Table 1:** Fuzzy rule table for $∆K_{p}$.

∆K_*p*_		*e*						
		NB	NM	NS	ZO	PS	PM	PB
*e_c_*	NB	PB	PB	PM	PM	PS	ZO	ZO
	NM	PB	PB	PM	PS	PS	ZO	NS
	NS	PM	PM	PM	PS	ZO	NS	NS
	ZO	PM	PM	PS	ZO	NS	NM	NM
	PS	PS	PS	ZO	NS	NS	NM	NM
	PM	PS	ZO	NS	NM	NM	NM	NB
	PB	ZO	ZO	NM	NB	NB	NB	NB

**Table 2:** Fuzzy rule table for ΔK_*i*_.

ΔK_*i*_		*e*						
		NB	NM	NS	ZO	PS	PM	PB
*e_c_*	NB	NB	NB	NM	NM	NS	ZO	ZO
	NM	NB	NB	NM	NS	NS	ZO	ZO
	NS	NB	NM	NS	NS	ZO	PS	PS
	ZO	NM	NM	NS	ZO	PS	PM	PM
	PS	NM	NS	ZO	PS	PS	PM	PB
	PM	ZO	ZO	PS	PS	PM	PB	PB
	PB	ZO	ZO	PS	PM	PM	PB	PB

**Table 3:** Fuzzy rule table for $∆K_{d}$.

∆K_*d*_		*e*						
		NB	NM	NS	ZO	PS	PM	PB
*e_c_*	NB	PS	NS	NB	NB	NB	NM	PS
	NM	PS	NS	NB	NM	NM	NS	ZO
	NS	ZO	NS	NM	NM	NS	NS	ZO
	ZO	ZO	NS	NS	NS	NS	NM	ZO
	PS	ZO	ZO	ZO	ZO	ZO	ZO	ZO
	PM	PB	NS	PS	NS	PS	PS	PB
	PB	PB	PM	PM	PM	PS	PS	PB

**Table 4:** Result of static test.

Group	Before Leveling		After Leveling		Leveling Time
	*α*	*β*	*α*	*β*	
Front highest	6.1°	1.1°	1.3°	0.4°	3.0 s
Rear highest	−5.1°	0.8°	−1.4°	0.5°	2.8 s
Left highest	8.3°	0.3°	1.3°	1.1°	3.0 s
Right highest	−8.2°	1.3°	−1.4°	0.7°	3.1 s
Left front highest	5.8°	3.1°	1.1°	1.0°	3.8 s
Left front highest	7.7°	−4.1°	1.0°	−1.4°	4.7 s
Right rear highest	−6.0°	−4.1°	−0.9°	−1.4°	3.7 s
Right front highest	4.1°	−4.1°	1.2°	−1.4°	4.5 s

**Table 5:** Comparison of related studies.

Index	This Study	Ref [35]	Ref [36]	Improvement
Control method	Fuzzy PID	QBP-PID	No leveling	No training required
Drive mode	Electric drive	Hydraulic drive	Electric drive	Electronic control
Dynamic response	Quick response(±1.5°)	Quick response(<1.5°)	No leveling	No training required

## Data Availability

The data used to support the findings of this study are included within the article.
